# Genome Characterisation of *Priestia megaterium* mj1212 and Its Synergistic Effect With N‐Acetylglucosamine in Enhancing Soybean Salt Stress Tolerance

**DOI:** 10.1111/pce.70093

**Published:** 2025-08-05

**Authors:** Sang‐Mo Kang, Ibrahim Khan, Sajjad Asaf, Byung‐Wook Yun, In‐Jung Lee

**Affiliations:** ^1^ Department of Applied Biosciences Kyungpook National University Daegu Republic of Korea; ^2^ Natural and Medical Science Research Center University of Nizwa Nizwa Oman

**Keywords:** antioxidants, genome sequencing, GlcNAc, mj1212, organic acids, phytohormones, SNO

## Abstract

As a sustainable alternative to inorganic fertilisers, the combined application of plant‐growth promoting microbes and organic amendments offers an efficient biological approach to enhance plant growth under stress conditions. In this study, we present the complete genome sequence and functional annotation of the soil bacterial strain *Priestia megaterium* mj1212 (formerly classified as *Bacillus megaterium*), supporting its recent taxonomic reclassification and revealing its potential for secondary metabolites production, plant growth promotion, and adoption to environmental stresses. Our results showed that the combined application of N‐acetylglucosamine (GlcNAc) and mj1212 exhibited a synergistic effect, significantly increasing the length and weight of soybean shoots and roots by up to 24.36% and 42.22%, and 10.95% and 14.51%, respectively, under 150 mM NaCl stress. In addition, both sole and combined treatments of GlcNAc and mj1212 significantly enhanced root nodules formation, photosynthetic parameters, and relative water contents. Similarly, the individual and combined treatments of GlcNAc and mj1212 significantly increased polyphenol oxidase and flavonoid content, while reducing superoxide oxidase and catalase (CAT) activities. Organic acids analysis revealed increased levels of citric acids, and malic acids, while succinic acids and lactic acids levels decreased significantly under both conditions, with a slight antagonistic effect observed under stress. Notably, nitric oxide (SNO) levels, which decreased by 60.59% under NaCl stress, were restored by 492.55% of the stress level (nM/µg) with the combined treatment of GlcNAc and mj1212. The findings also suggested that GlcNAc and mj1212 treatments could improve soybean tolerance to salt stress by modulating abscisic acids biosynthesis. These findings underscore the potential of mj1212 and GlcNAc as effective biofertilizers for enhancing salt stress tolerance and promoting sustainable crop growth.

## Summary statement

Complete genome of *P. megaterium* mj1212 was sequenced, supporting its reclassification and revealing functions in stress adaptation and plant growth promotion. Combined treatment with GlcNAc and mj1212 increased soybean shoot and root growth by up to 42.22% under salt stress. GlcNAc and mj1212 treatments enhanced root nodules, photosynthetic efficiency and water content, improving salt stress resilience. PPO and flavonoid levels increased, while antioxidant enzymes like SOD and CAT decreased with GlcNAc and mj1212 treatments. Combined treatment of GlcNAc and mj1212 restored nitric oxide levels and modulated ABA biosynthesis to enhance salt tolerance.

## 1 Introduction

Salinity stress significantly inhibits plant growth and development by disrupting essential biochemical processes, resulting in reduced water content, decreased photosynthetic efficiency and nutrient imbalances (I. Khan et al. 2021). In plants, including soybean, salinity stress induces osmotic and ionic imbalances, which in turn stimulate the production of reactive oxygen species (ROS) such as hydrogen peroxide (H_2_O_2_), superoxide (O_2_^−^), singlet oxygen (^1^O_2_) and the hydroxyl radical (OH) (M. Khan et al. 2021). Although ROS production is a natural part of plant cellular metabolism, under stress conditions, their excessive accumulation can damage lipids, proteins, carbohydrates and nucleic acids, ultimately impairing key functions like photosynthesis and nutrient uptake (Hasanuzzaman et al. 2021). ROS can act as either signalling molecules or stressors, depending on the crucial equilibrium between their generation and scavenging (Rahman et al. 2024). Oxidative stress increases under abiotic stresses, including salinity, due to disruption of the balance between ROS production and antioxidant defences. Both enzymatic antioxidants, such as catalase (CAT), superoxide dismutase (SOD), glutathione peroxidase, ascorbate peroxidase and peroxidase (I. Khan et al. 2024), as well as non‐enzymatic antioxidants like glutathione (GSH), ascorbic acid (AsA), tocopherols, carotenoids, polyphenol and flavonoids, contribute significantly to antioxidant defence (Sachdev et al. 2021; Tewari et al. 2021). Furthermore, organic acids (OAs) such as citric acids (CAs), malic acids (MAs), succinic acids (SAs) and lactic acids (LAs) are essential for primary metabolic pathways, such as the tricarboxylic acid (TCA) cycle, which helps plant growth, photosynthesis, energy production and pH regulation, especially under stress conditions (Farhad et al. 2023; Peng et al. 2023). Under stress conditions, plants exude OAs at the root‐soil interface, facilitating nutrient uptake and improving tolerance to toxic metals. Compared to other acid types, such as amino acids, OAs provide greater versatility in mitigating stress effects and function as efficient chelating agents. In addition, the exudation of root OAs contributes to the enhancement of soil carbon sequestration (Ahlawat et al. 2024; Panchal et al. 2021). Signalling pathways involving calcium ions (Ca²⁺), nitric oxide (NO) and phytohormones such as abscisic acid, salicylic acid (SA), ethylene (ET) and jasmonic acid (JA) regulate antioxidant production by modulating stress‐responsive gene expression and maintaining Na^+^ and Cl^‐^ ion homoeostasis (Mir et al. 2024; Raza et al. 2022).

Soybean (*Glycine max*) is widely used in plant stress physiology studies, particularly under salt stress, due to its global agricultural significance as a high‐protein and oil‐yielding crop. Its moderate salt‐sensitivity makes it an ideal model for studying salt stress, as it allows for clear observations of physiological and molecular responses, including ion homoeostasis, antioxidant activity and osmolyte accumulation (Cheng et al. 2020). The availability of a fully sequenced genome and diverse germplasm collection facilitates genetic and transcriptomic studies, while its adaptability to controlled environments and the development of genetic tools support experimental manipulation and functional gene studies (Li et al. 2023). In addition, soybean‐growing regions are often affected by soil salinity, making it a relevant model for addressing real‐world agricultural challenges (C. Feng et al. 2023). Traditional breeding, modern genetic modification and alternative phytoremediation strategies such as using salt‐accumulating halophytes, chemical amendments and soil washing have been explored to improve salt tolerance in crops; however, but their large scale application remains limited due to high costs, long developmental periods, land use demands and potential environmental or soil structure impacts (Z. Liu et al. 2025; van den Burg et al. 2024). In this regard, in situ stabilisation using bio‐organic amendments and beneficial microbes offers a cost‐effective and sustainable solution for remediating salt‐contaminated arable soils, making it an attractive alternative to conventional strategies (Bello et al. 2021; I. Khan, Asaf, Kang 2025).

N‐acetylglucosamine (GlcNAc) is a vital monosaccharide involved in various biological processes in both microbial and plant systems, especially in microbial interactions, and serves as an important source of carbon and nitrogen for several microbial species (Katz 2019; Xu et al. 2022). It is an essential component of bacterial cell walls, contributing to structural integrity, supporting cell division, and providing protection against environmental stresses such as osmotic pressure and antimicrobial agents (Roy et al. 2023). It significantly contributes to their ability to adhere to surfaces, establish infections, and evade immune responses by forming a protective barrier around the bacterial colony (Ansari et al. 2022; Vuong et al. 2004). In addition, GlcNAc functions as a signalling molecule within microbial communities, modulating quorum sensing pathways and regulating the expression of genes associated with virulence and biofilm formation (Min and Park 2025). In plants, GlcNAc promotes growth and resilience through various mechanisms, including promoting cell elongation and division, improving root and shoot development and increasing tolerance to abiotic stress by boosting protective compounds and metabolic changes. It also increases nutrient absorption, photosynthetic efficiency and chlorophyll content, resulting in improved plant health and productivity (Kang et al. 2024). However, the role of GlcNAc in stress alleviation should be considered alongside other well‐established organic amendments, such as humic acid and silicon, which have been more extensively studied for their effects on salt stress tolerance through enhanced antioxidant activity, osmotic regulation and improved nutrient uptake (Abbas et al. 2022; Abu‐Ria et al. 2023; Aouz et al. 2023). In addition, O‐GlcNAc activates plant defence mechanisms, enhancing resistance to stress conditions, ultimately resulting in higher yields and improved stress resistance, making it a valuable tool for sustainable agriculture (Xue et al. 2024). GlcNAc also plays a crucial role in plant hormone biosynthesis and signalling pathways, directly influencing plant growth, development and stress responses by acting as a key regulator through a process called O‐GlcNAcylation. In this process, GlcNAc attaches to specific amino acids on proteins, modifying their function and affecting downstream signalling cascades (Chen et al. 2022; B. E. Lee et al. 2021). Furthermore, changes in O‐GlcNAc levels can affect the production of NO by regulating the enzyme iNOS, which in turn impacts processes such as inflammation and cellular stress responses (Abramowitz and Hanover 2022).

In addition to the inherent defence mechanisms of plants, plant growth promoting (PGP) bacteria, possess specialised mechanisms that play a crucial role in salt stress tolerance and plant growth promotion (Numan et al. 2018). These bacteria enhance plant growth and soil fertility by decomposing organic matter, enhancing water uptake, dissolving nitrogen, phosphorus and potassium, promoting symbiotic relationships, inhibiting pathogens and inducing plant resistance (Wang et al. 2024). These bacteria have been extensively studied for their production of various metabolites, including indole‐3‐acetic acids (IAA), jasmonic acids (JA), gibberellins (GA), OAs, amino acids, siderophores and many others (Kwon et al. 2024).

Complete replacement of inorganic fertilisers is a significant challenge; therefore, in the current study, two biostimulants, GlcNAc and the endophytic beneficial bacterial strain, *Priestia megaterium* (mj1212), were selected to evaluate their individual and combined potentials in promoting growth and alleviating salt stress in soybean plants. Our previous studies demonstrated that *P. megaterium* mj1212, isolated from rhizosphere soil and formerly classified as *Bacillus megaterium* (J. M. Liu et al. 2023; Patel and Gupta 2020), possesses significant potential as a plant growth promoter. It enhances the growth of mustard plants under controlled conditions and improves the growth of alfalfa (*Medicago sativa*) under drought stress through the production of OAs and its phosphate‐solubilising capacity. In addition, *P. megaterium* strains are known to produce phytohormones such as indole‐3‐acetic acids (IAA), secrete siderophore for iron acquisition, exhibit strong phosphate solubilising capacity and produce ACC deaminase to lower plant ethylene levels under stress (Lin et al. 2022; Thakur et al. 2024). The interaction between *B. megaterium* mj1212 and crops such as mustard and alfalfa has been shown to significantly alter plant biochemical pathways, enhancing chlorophyll content, modulating the antioxidant system and endogenous ABA biosynthesis, and increasing the levels of glucose, fructose, sucrose and amino acids (Kang et al. 2021; Kang et al. 2014). Notably, in alfalfa, mj1212 improved drought tolerance, while in mustard, it enhanced nutrient uptake and growth under controlled conditions. Based on these findings, we hypothesised that this bacterial strain could effectively alleviate salt stress in plants such as soybean by modulating the antioxidant system, enhancing the production of OAs and phytohormones like ABA, and by developing an eco‐friendly phytoremediation strategy to efficiently mitigate salt stress and promote soybean growth. As part of this study, we performed whole‐genome sequencing and functional annotation of mj1212 to better understand its genetic potential for plant growth promotion and stress alleviation. To achieve this, we conducted an in‐depth analysis at the genomic, physiological and biochemical levels to comprehensively understand the impact of this rhizospheric soil bacterial strain on soyabean plant growth and stress resilience under salt stress conditions. The results of this study have the potential to contribute to the advancement of phytoremediation strategies and the promotion of sustainable agricultural practices by alleviating salinity stress and enhancing the growth of healthy crops in both controlled and high salinity conditions.

## 2 Methodology

### 2.1 Whole Genome Sequencing Functional Annotations of mj1212

Genomic DNA was extracted from a 24‐h culture of *P. megaterium* mj1212 grown at 28°C in nutrient broth (NB), following the manufacturer's instructions (Genomic DNA Purification Kit; Promega, USA). In brief, the culture was centrifuged at 10 000 rpm for 10 min at 4°C, and the resulting bacterial pellet was washed repeatedly with phosphate‐buffered saline (PBS) until the wash solution became clear. DNA concentration and purity were measured using a Nanodrop 2000 UV‐Vis spectrophotometer (Thermo Fisher Scientific, USA) and a Qubit fluorometer 2.0 (Invitrogen, USA). The whole‐genome sequencing of the mj1212 was performed at the Next Generation Sequencing core facility of the Kyungpook National University, Republic of Korea, using a hybrid approach combining Illumina NovaSeq. 6000 and Oxford Nanopore MinION platforms. For Illumina sequencing, paired‐end libraries were prepared; for nanopore sequencing, long‐read libraries were prepared using the Ligation Sequencing Kit. Raw Illumina reads were quality‐filtered using trimmomatic, and nanopore reads were base‐called and quality‐checked using Guppy V5.0. The hybrid de novo genome assembly was performed using Unicycler, which integrates short and long reads to improve assembly continuity and accuracy. The Prokaryotic Genome Annotation Pipeline and Rapid Annotation using Subsystem Technology were employed to functionally annotate the assembled genome and identify key genes associated with plant growth promotion and metabolic pathways involved in mitigating and tolerating salt stress. The complete genome sequence of *P. megaterium* MJ112 has been deposited in the TBL/EMBL/GenBank databases under the BioProject number PRJNA1248167, BioSample number SAMN47852027 and the accession numbers CP187221‐CP187225.

#### 2.1.1 Average Nucleotide Identity (ANI) Analysis

To determine the species affiliation of the mj1212 genome, we use the command‐line bit to retrieve representative genomes from 10 identified genera available in the NCBI database (M. Lee 2022). ANI values were calculated to perform pairwise nucleotide‐level comparisons using FastANI v.1.133, a past alignment‐free computational tool. The novel species was used as the query against other genomes. An all‐vs‐all ANI analysis was then performed, and the results were visualised with ANIclustermap v.1.2.0.

#### 2.1.2 Analysis of Metabolic System

The carbohydrate active enzymes (CAZy) database was used to investigate the functional diversity of CAZymes in the mj1212 strain, with a particular focus on the diversity of enzymes involved in the synthesise, modification and degradation of carbohydrates.

### 2.2 Seed Treatment and Germination

Soybean seeds of the Hannam cultivar were obtained from the Genetic Research Center at Kyungpook National University, South Korea. The seeds were thoroughly surface sterilised using 70% ethanol, followed by 2.5% sodium hypochlorite (NaOCl), and then washed twice with double‐distilled water (ddH2O). The sterilised seeds were transferred to Petri dishes, added with 5 mL of dH_2_O, and placed in a germination chamber in the dark at room temperature. After proper germination, uniform‐sized seedlings at the V1 stage (one unrolled trifoliate leaf) were selected and transplanted into plastic pots (10 cm × 9 cm) containing autoclaved sandy loam soil.

### 2.3 Experimental Design and Treatments

The plants were categorised into the following treatment groups: (ⅰ) Control, dH_2_O‐irrigated; (ⅱ) GlcNAc‐treated; (ⅲ) mj1212‐treated; (ⅳ) GlcNAc+mj1212‐treated; (ⅴ) salt stress, irrigated with 150 mM NaCl solution; (ⅵ) GlcNAc+NaCl‐treated; (ⅶ) mj1212+ NaCl‐treated; and (ⅷ) GlcNAc+mj1212+ NaCl‐treated. The pots were kept in a greenhouse under a 14/10 h of light/dark cycle, with 65%–70% relative humidity, at a temperature of 24°C–28°C, and an illuminance of 1000 Em^2^ using fluorescent bulbs. The experiment was conducted with five replicates per treatment, and each assay was performed with three technical replicates. Control plants were watered with dH_2_O every 12 h, while plants subjected to salt stress received 20 mL of 150 mM NaCl solution every second day, altering with dH_2_O. To assess and compare the salt stress tolerance and growth‐promoting potentials of GlcNAc and mj1212, the designated plant groups were drenched with 50 mL of 1 mM GlcNAc solution (prepared by dissolving 221.21 mg of GlcNAc in 1 L of dH_2_O) and 50 mL of mj1212 suspension. The mj1212 suspension, harvested after 5 days of cultivation in the logarithmic (log) growth phase, was applied directly to the soil near the root zone.

### 2.4 Exponential Growth Measurement of mj1212 Under Varying NaCl and GlcNAc Concentrations

The exponential growth rate was measured by growing the mj1212 strain in 250 mL tryptic soy broth medium supplemented with 20 mM‐80 mM GlcNAc, each with 5% and 10% NaCl concentrations. As a control mj1212 was also grown without NaCl and GlcNAc. All the flasks were incubated for 48 h at 28°C in a shaking incubator at 150 rpm. Samples were taken, and reading were carried out at 650 nm.

### 2.5 Preparation of Bacterial Inoculum

A sterile inoculating loop was used to transfer the mj1212 culture into 500 mL conical flasks containing nutrient broth (NA) media. After inoculating, the flasks were incubated at 28°C on a rotatory shaker at 150 rpm for 5 days, and then bacterial biomass (pellets) was separated from the liquid medium (supernatant) by centrifugation. Subsequently, 100 mL of the collected culture, at a concentration of 10^8^ cells/mL (OD_600_ nm 0.2, determined based on a standard curve generated by corelating OD_600_ reading with plate counts (CFU/mL), was inoculated into the respective treatment groups of soybean plants, following the experimental design procedure.

### 2.6 Plants Harvesting and Assessment of Growth Attributes

Plants were exposed to their respective treatments for 20 days. Afterwards, they were harvested, and growth attributes, including shoot and root length, biomass and the number of root nodules, were measured. The samples were then immediately frozen in liquid nitrogen and stored at −80°C for further analyses.

### 2.7 Assessment of Photosynthetic Components

Chlorophyll content was assessed according to the method outlined by I. Khan et al. (2024). In brief, 200 mg of freshly ground samples from each treatment group were homogenised in 80% acetone, briefly vortexed, and then centrifuged at 15 000 rpm at 4°C for 10 min. The absorbance of the supernatant was measured spectrophotometrically at 663 and 645 nm to quantify chlorophyll‐a and chlorophyll‐b, respectively. Total chlorophyll content was calculated using the formula: Total chlorophyll (mg/g DW) = [(20.2 × A645) + (8.02 × A663)/100 × W] × V. Chlorophyll fluorescence was measured using a chlorophyll fluorometer (OS5p+ ; Opti‐Sciences, USA), with specific fluorescence parameters analysed as shown in Table S5. The net photosynthetic rate [Pn, μmol/(m^2^·s)] and transpiration rate [E, mmol/(m^2^·s)] were also measured in situ on the third or fourth fully expanded leaf, counted from the apex of the new shoots.

### 2.8 Assessment of Relative Water Content (RWC)

To determine the RWC of leaves, the method described by I. Khan et al. (2025) was used. Fully expanded levels from the third node, counting back from the apex of new shoots, were collected in triplicate during the morning. The fresh weight (FW) of each leaf was measured immediately after collecting using an analytical balance. To achieve full turgidity, the leaves were immersed in dH_2_O in Petri dished for 8 h. After gently blotting to remove surface moisture, the turgid weight (TW) was measured. Subsequently, the leaves were dried in an oven at 70°C for 48 h, and their dry weight (DW) was measured. The RWC was calculated using the formula: RWC = (TW − DW)/(FW − DW) × 100.

### 2.9 Assessment of Antioxidants

To assess polyphenol oxidase (PPO) activity, the previously established protocol (M. Khan et al. 2021) was used with slight modification. In brief, finely ground samples (200 mg) from each treatment group were homogenised in 100 mM phosphate buffer (pH 6.8), briefly vortexed, and then incubated at room temperature for 1 h. The homogenates were then centrifuged at 10 000 rpm for 10 min, and 50 μL of the resulting supernatants were carefully transferred to new Eppendorf tubes. To each tube, 50 μL of pyrogallol and 100 μL of 100 mM phosphate buffer were added. The absorbance was then measured at 420 nm using a spectrophotometer.

For the assessment of total flavonoid content, the method described by R. Ali et al. 2022) was followed with slight modifications. Pulverised samples (200 mg) were mixed with 1.5 mL of 80% acetone, vortexed briefly, and centrifuged to obtain a clear supernatant. In a 96‐well microplate, 50 μL of the supernatant was mixed with 50 μL of 10% aluminium chloride (AlCl_3_) and 50 μL of 1 M NaOH. dH_2_O was then added to adjust the final volume to 200 μL per well. Following a 30 min incubation at room temperature, absorbance was measured at 415 nm using a spectrophotometer.

For the quantification of SOD, the method described by Peter et al. (2024) was followed with modifications. Briefly, 200 mg of the samples were homogenised in 5 mL of extraction buffer containing 50 mM of Tris‐HCl and 10 mM of EDTA, followed by 30 min sonication. The mixture was then centrifuged at 10 000 rpm for 10 min. The resulting supernatants were used to prepare three fractions: (a) 50 μL of supernatant mixed with 150 μL of extraction buffer and 50 μL pyrogallol, (b) 50 μL supernatant mixed with 200 μL of extraction buffer, and (c) 150 μL of extraction buffer mixed with 100 μL of pyrogallol. Absorbance was measured at 420 nm, and SOD activity was calculated using the formula: SOD U/mg= [1 − (A − B)/C) × 100], where A absorbance of the first fraction, B is the absorbance of the second fraction and C is absorbance of the third fraction.

For the catalase (CAT) assay, our previously established protocol (Aizaz et al. 2023) was followed. Each sample was homogenised in 1.5 mL of extraction buffer, prepared by dissolving Tris‐HCl, MgCl_2_, EDTA and PVP in dH_2_O while stirring at 35°C. After vortexing and centrifugation at 10 000 rpm for 10 min, 50 μL of the resulting supernatant was mixed with phosphate buffer and 0.2 M of H_2_O_2_ in a 96‐well plate. The absorbance was then measured at 240 nm.

### 2.10 Assessment of OAs Metabolites

For the assessment of OAs, including CAs, MAs, SAs and LAs, previously established protocols (S.‐B. Lee et al. 2020) were followed with slight modification. Briefly, 200 mg of sample from each treatment group was finely ground in liquid nitrogen and extracted with 0.1 M HCl. After shaking for 15 min, the mixtures were incubated in a water bath for 15 min and then centrifuged at 8000 rpm. The supernatant was passed through a 0.22 μm syringe filter (Millipore, Billerica, MA, USA) before injecting 20 μL into the HPLC system. The analysis of OAs was performed using Shimadzu Prominence HPLC system equipped with a refractive index detector (RID‐10A). Separation was achieved on a PL Hi‐Plex H column (7.7 mm ID, 300 mm length) at a column temperature of 65°C. The mobile phase consisted of 0.005 M sulphuric acid (H_2_SO_4_) in water, delivered at a flow rate of 0.6 mL/min. The relative contents of OAs were quantified by calculating the ratio of each peak area to that of the internal standard and used for statistical analysis.

### 2.11 Assessment of S‐Nitrosothiol (SNO)

SNO concentrations were measured using a Sievers NOA‐280i Nitric Oxide Analyser (Estero, FL, USA), following a published protocol (D.‐S. Lee et al. 2024). Fresh soybean shoots (200 mg) were ground finely in liquid nitrogen and homogenised in PBS buffer (pH~7.4). After centrifugation at 14 000 rpm for 10 min at 4°C, the supernatants were transferred to fresh Eppendorf tubes. Protein concentration was quantified using the Bradford assay with Coomassie dye, and absorbance was measured at 595 nm using a UV spectrophotometer. Extracted proteins (100 μL) were injected into the analyser's reaction vessel containing a CuCl/cysteine reducing agent, with peak values recorded. The SNO levels (expressed as nM/μg of protein weight) were calculated using a CysNO‐mediated standard curve. In addition, the GPS‐SNO 1.0 software was used to predict and identify cysteine residues susceptible to S‐nitrosylation.

### 2.12 Assessment of Endogenous Abscisic Acids (ABA)

The method described by I. Khan et al. (2025) was used to assess ABA levels. In brief, freeze‐dried samples of each treatment group were treated with an ABA extraction solution consisting of isopropanol and acetic acid (95:5, v/v). To the resulting suspension, 100 ng of an ABA standard was added. The extraction process involved several steps: dichloromethane (CH_2_Cl_2_) was used to remove chlorophyll, followed by extraction with ethyle acetate (EtOAc). The upper layer was carefully transferred to fresh round‐bottom flasks and evaporated using a rotatory evaporator. The dried residues were washed with phosphate buffer, mixed with polyninylpyrrolidone (PVP) and the pH was adjusted to 2.5–3.5. A final extraction was then performed with EtoAc, and the resulting extract was dried under nitrogen (N_2_). The dried samples were then methylated using diazomethane, redissolved in dichloromethane and injected into a gas chromatography‐mass spectrometry/selective ion monitoring (GC‐MS/SIM) system (689N Network GC System and 5973 Network Mass Selective Detector; Agilent Technologies, Santa Clara, CA, USA). Quantification of ABA content was done by evaluating the peak areas corresponding to the ions at m/z 190 and 194.

### 2.13 Statistical Analysis

Data analysis and graphical visualisation for the various measurements and assays were performed using GraphPad Prism. Microsoft Excel was used to calculate mean values and standard deviation. Statistical significance among treatments was determined at *p* < 0.05 using Duncan's multiple range test and analysis of variance, both conducted in Statistical Analysis Software (SAS, version 9.1, Cary, NC, USA).

## 3 Results

### 3.1 Genome Sequencing and Functional Annotations

**Figure 1:**
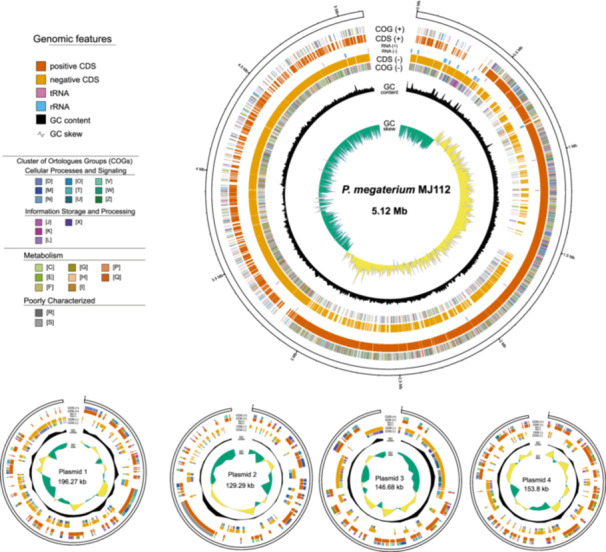
Circular genome maps of *P. megaterium* mj1212 and its four plasmids, showing coding sequences (CDS), RNA genes, GC contents, GC skew and COG functional classifications.

**Figure 2:**
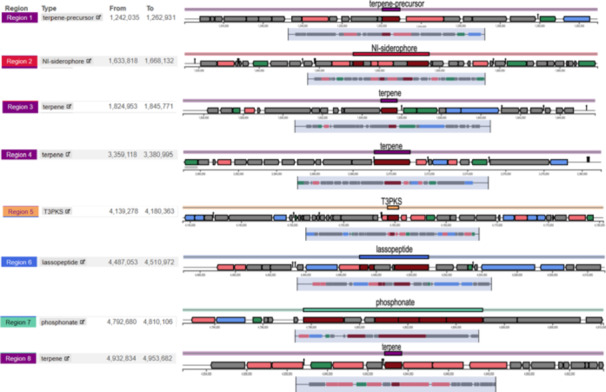
Predicted biosynthetic gene clusters (BGCs) in *P. megaterium* mj1212 genome, showing regions involved in terpene, siderophore, polyketide, lassopeptide and phosphonate biosynthesis. [Color figure can be viewed at wileyonlinelibrary.com]

The complete genome of *P. megaterium* mj1212 consists of a 511 7591 bp (5.12 Mb) circular chromosome with a G + C content of 38.3%, and four circular plasmids of 196 267 bp (196.27 kb), 129 292 bp (129.29 kb), 146 684 bp (146.68 kb) and 153 797 bp (153.8 kb), with G + C contents of 33.6%, 33.5%, 34.1% and 34.3%, respectively (Figure 1). When combined, the chromosome and plasmids contained 6290 annotated genes, including 121 tRNA, 40 complete rRNA, 7 ncRNA and 6122 protein‐coding sequences (CDSs; Table S1). Eight biosynthetic gene clusters were identified in the mj1212 genome using the antiSMASH, including four terpene clusters, one NI‐siderophore cluster, one type Ⅲ polyketide synthase cluster, one lassopeptide cluster and one phosphonate cluster. Comparative analysis showed similarities with known BGCs, but some clusters were divergent, suggesting potential for new metabolic discovery (Figure 2). Our main objective was to specifically annotate the genes involved in plant‐growth‐promoting rhizobacteria (PGPR) traits. Our analysis revealed that 32 genes are significantly associated with various PGPR traits including phosphate metabolism, IAA production, osmotic stress response, siderophore production and interaction with the host plant (Table S2).

#### 3.1.1 Analysis of Metabolic System of *P. megaterium* mj1212 Genome

**Figure 3:**
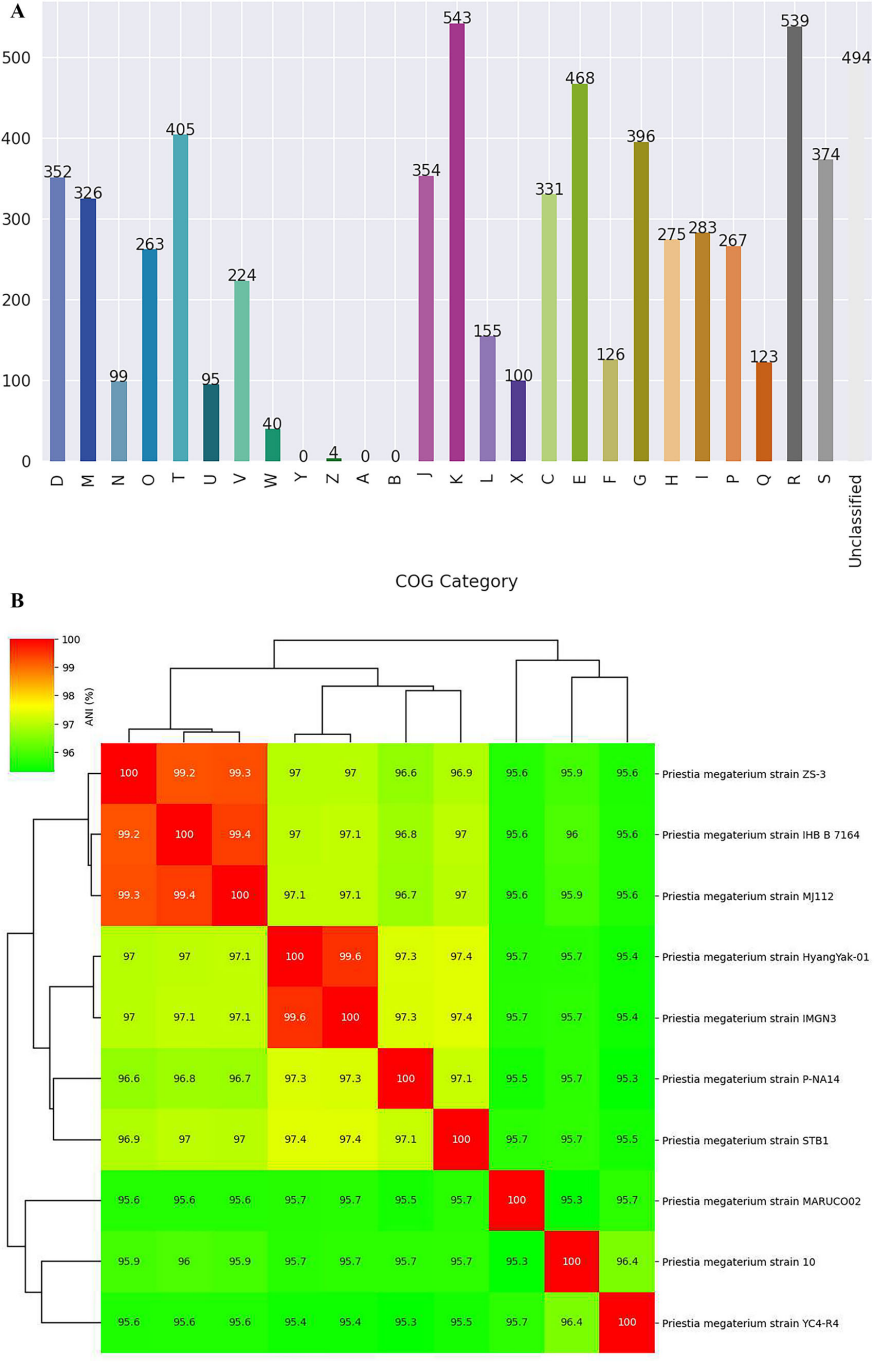
(A) Distribution of coding sequences across COG categories in the *P. megaterium* mj1212 genome. (B) Average nucleotide identity (ANI) heatmap showing genomic similarity among *P. megaterium* strains, with hierarchical clustering based on pairwise ANI values. [Color figure can be viewed at wileyonlinelibrary.com]

The study comprehensively explores the biological functions and characteristics of genes in *P. megaterium* mj1212 through genome metabolic system analysis. The Cluster of Orthologous Groups analysis revealed the presence of PGP and stress resistance genes. Notably, genes associated with cellular processes and signalling such as intracellular trafficking, defence mechanisms and extracellular structures formation as well as information storage and processing, including translation, transcription and replication. In addition, the annotation indicated the presence of genes involved in the transport of amino acids, lipids, nucleic acids and inorganic ions (Figure 3A and Table S3). The CAZy results of the mj1212 genome show that there are 145 genes coding carbohydrate active enzymes, revealing that they can metabolise a variety of carbohydrates (Table S4). Among them, 45 genes were predicted to encode glycosyltransferases, 24 encoded carbohydrate esterases, 30 encoded glycosyltransferases, 40 encoded carbohydrate binding lyases, 4 encoded polysaccharide lyases and only 2 genes were associated with auxiliary activities.

#### 3.1.2 Taxonomic Identification of *P. megaterium* mj1212

ANI analysis of 10 *P. megaterium* strains revealed high genomic similarity among several groups. Strains ZS‐3, IHB B 7164 and mj1212 showed >99% ANI, indicating close relatedness. Similarly, HyangYak‐01 and IMGN3 formed a distinct cluster with >99% ANI. In contrast, strains MARUCO02, 10 and YC4‐R4 exhibited lower ANI values (~95%–96%), suggesting greater genomic divergence. The results showed both conserved and divergent genomic relationships within the *P. megaterium* strains (Figure 3B).

### 3.2 NaCl and GlcNAc Concentrations Affect Exponential Growth of mj1212

To investigate the effects of salinity and GlcNAc on the growth dynamics, mj1212 was grown in PSB medium containing different GlcNAc and GlcNAc concentrations, and the growth rate was estimated (Figure S1). As expected, under no NaCl condition, the growth rate increased with increasing GlcNAc concentration up to 40 mM. However, beyond this concentration, growth declined significantly and became lower than the growth observed with no GlcNAc at 80 mM GlcNAc. Under 5% NaCl, the highest growth was observed with 20 mM GlcNAc, followed by a decline at higher GlcNAc concentrations, similar to control. In contrast, under 10% NaCl, the highest growth was observed at 60 mM GlcNac.

### 3.3 GlcNAc and mj1212 Improve Plant Growth Under Salt Stress

The soybean plants were treated according to the experimental design, which included individual and combined applications of GlcNAc and mj1212 under NaCl stress. The results shown in Table 1 indicate that under non‐stress conditions, GlcNAc and mj1212 significantly increased shoot length by 6.05% and 8.6%, respectively, while their combined application resulted in a 14.01% increase in shoot length. Similarly, under NaCl stress, shoot length increased significantly by 12.72% and 15.27% when soybean plants were treated with GlcNAc and mj1212, respectively, and their combined application resulted in a 24.36% increase compared to the NaCl‐treated plants. Similar results were also observed on root length of soybean plants both under control and NaCl stress conditions. Under non‐stress conditions, GlcNAc and mj1212 treatments significantly increased shoot and root weight by up to 8.21% and 14.51%, respectively, when applied individually, and by 10.95% and 14.51% respectively, when applied in combination. Moreover, under stress conditions, the lowest number of root nodules was found in the control (NT), followed by mj1212 (with a 5.19% increase), while GlcNAc treatment resulted in the highest increase (9.84%). Under NaCl stress conditions, the highest increase (94.13%) in root nodules was recorded in GlcNAc‐treated plants, followed by GlcNAc + mj1212‐treated plants (85.71%), while individual treatment of mj1212 resulted in a 73.26% increase.

**Table 1:** Effect of GlcNAc, *P. megaterium* mj1212 and their combination on shoot length, root length, shoot weight, root weight, number of root nodules and chlorophyll content under non‐stress and salinity stress conditions.

	Shoot length	Root length	Shoot weight	Root weight	Number of root nodules	Chlorophyll content
None stress						
NT	31.4 ± 0.67d	30.6 ± 0.77de	7.3 ± 0.14cd	6.2 ± 0.06b	57.7 ± 7.33a	363.3 ± 47.63ab
GlcNAc	33.3 ± 0.59bc	32.4 ± 0.83bc	7.5 ± 0.15bc	6.6 ± 0.15ab	64.0 ± 9.02a	458.7 ± 33.51a
MJ1212	34.1 ± 0.32ab	33.8 ± 0.21ab	7.9 ± 0.08ab	7.1 ± 0.31a	60.7 ± 2.85a	405.3 ± 35.02a
GlcNAc + MJ1212	35.8 ± 0.49a	34.9 ± 0.30a	8.1 ± 0.05a	7.1 ± 0.20a	62.0 ± 3.61a	416.3 ± 13.78a
Salinity stress						
NT	27.5 ± 0.53e	27.6 ± 0.35f	6.1 ± 0.26d	4.5 ± 0.15d	27.3 ± 8.21b	299.3 ± 31.37b
GlcNAc	31.0 ± 0.68d	29.7 ± 0.31e	7.2 ± 0.08c	5.4 ± 0.16c	53.0 ± 7.57a	357.0 ± 35.04ab
MJ1212	31.7 ± 0.81cd	31.6 ± 0.31cd	7.5 ± 0.12bc	6.1 ± 0.30b	47.3 ± 7.62ab	373.7 ± 32.71a
GlcNAc + MJ1212	34.2 ± 0.47ab	34.5 ± 0.48a	7.6 ± 0.15abc	6.4 ± 0.13b	50.7 ± 8.74a	376.0 ± 9.71a

### 3.4 GlcNAc and mj1212 Enhance Chlorophyll Content and Photosynthetic Parameters

**Figure 4:**
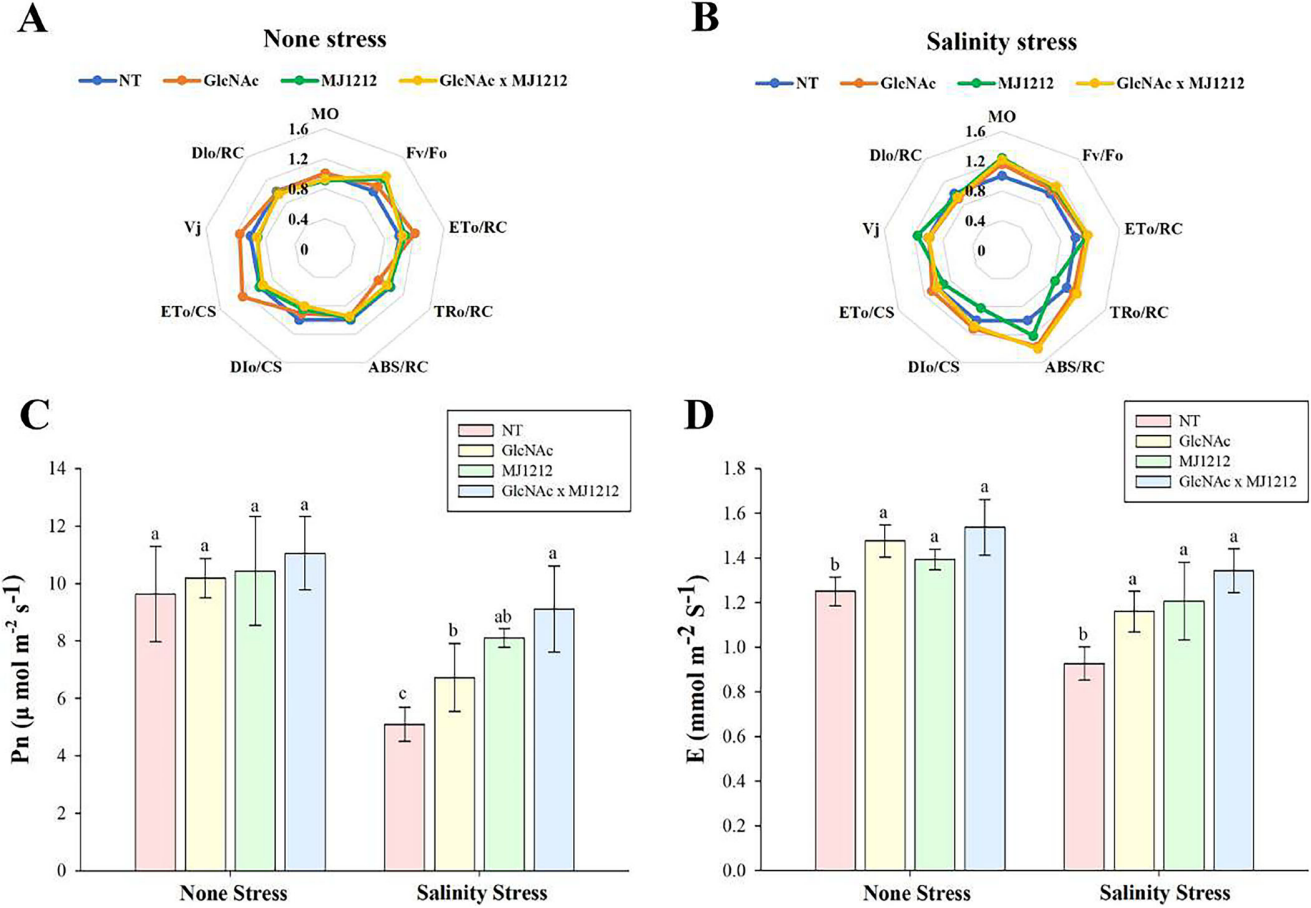
Photosynthetic parameters measured using the OJIP curve under normal (A) and salinity stress (B) conditions, and the parameters net photosynthetic rate (Pn) and transpiration rate (E) measured under normal (C) and salinity stress (D) conditions, in response to GlcNAc, *P. megaterium* mj1212, and their combination. Each bar represents the mean of three experimental replicates, with error bars indicating the standard error of the mean. Different letters above the bars indicate significant differences between treatments at *p* < 0.05, as determined by DMRT. [Color figure can be viewed at wileyonlinelibrary.com]

The results revealed that both GlcNAc and mj1212 significantly enhance chlorophyll content under both non‐stress and NaCl stress conditions. As shown in Table 1, under non‐stress conditions, the individual application of GlcNAc resulted in a 26.25% increase in chlorophyll content compared to the non‐treated control. Similarly, treatment with mj1212 led to an 11.56% increase, while the combined application of GlcNAc and mj1212 resulted in a 14.58% increase compared to the control. Under NaCl stress, the control plants exhibited chlorophyll content of 299.3 mg/g DW, which increased by 19.27%, 24.85% and 25.62%, when treated with GlcNAc, mj1212 and their combined application, respectively. Evaluating quantum yield and stress levels in crops is essential, and this can be effectively achieved by assessing photosynthetic parameters through OJIP curves. Under non‐stress conditions, GlcNAc treatment enhanced electron transport flux (ET_o_/CS) by 25.89% and the efficiency of the water‐splitting complex on the donor side of PSⅡ (ET_o_/RC) by 20.79% (Figure 4A). Similar trends were observed in NaCl‐stressed plants, though with slight variations. The individual application of mj1212 led to a slight increase in relative variable fluorescence (Vj) by 15.19% and absorbed photon flux per PSⅡ reaction centre (ABS/RC) by 21.9%, while trapped energy flux per PSⅡ reaction centre (TR_o_/RC) decreased significantly by 18.1% (Figure 4B). Furthermore, the combined application of GlcNAc and mj1212 significantly increased the net rate of PSⅡ closure (M_o_), and ABS/RC by 21.7% and 39.99%, respectively, compared to NaCl stressed plants. Table S5 provides a list of abbreviations used in the OJIP curves. As shown in Figure 4C, the net photosynthetic rate (Pn) exhibited a notable increase in response to GlcNAc and mj1212 treatments, with the most pronounced enhancement observed under their combined application in both under control and NaCl stress conditions. In individual treatments, mj1212 showed better effect compared to GlcNAc, increasing Pn by up to 8.41% and 58.99% under control and stress conditions, respectively. Notably, the combined application of GlcNAc and mj1212 resulted in a further increase of up to 14.81% and 78.87% under control and stress conditions, respectively. A similar trend was also observed in the transpiration rate (E), except for the individual treatment of GLcNAc, which enhanced E by up to 18.13%, whereas mj1212 increased it by up to 11.46%. Under stress conditions, the combined application of GlcNAc and mj1212 further increased E by up to 44.95% (Figure 4D).

### 3.5 GlcNAc and mj1212 Treatments Improve RWC Under Salt Stress

Maintaining cellular water balance is crucial for plant adaptation to stress conditions. In soybean plants subjected to NaCl stress, RWC significantly decreased, indicating a reduction in their water retention capacity. However, the application of GlcNAc, mj1212 and their combination effectively improved leaf RWC under both stress and non‐stress conditions. When comparing the effects of mj1212 inoculation and GlcNAc treatment, it is evident that GlcNAc demonstrated a slightly greater ability to improve RWC under NaCl stress, increasing it by 12.97%, while mj1212 resulted in a 12.38% increase compared to NaCl‐stressed plants. The combined application of GlcNAc and mj1212 resulted in the highest RWC, with a 16.01% improvement compared to their NaCl‐stressed counterparts. These findings indicate that these treatments contribute to improving water retention in plant cells, thereby mitigating the detrimental effects of NaCl stress (Figure S2).

### 3.6 Effects of GlcNAc and mj1212 on Antioxidant Activities Under NaCl Stress

**Figure 5:**
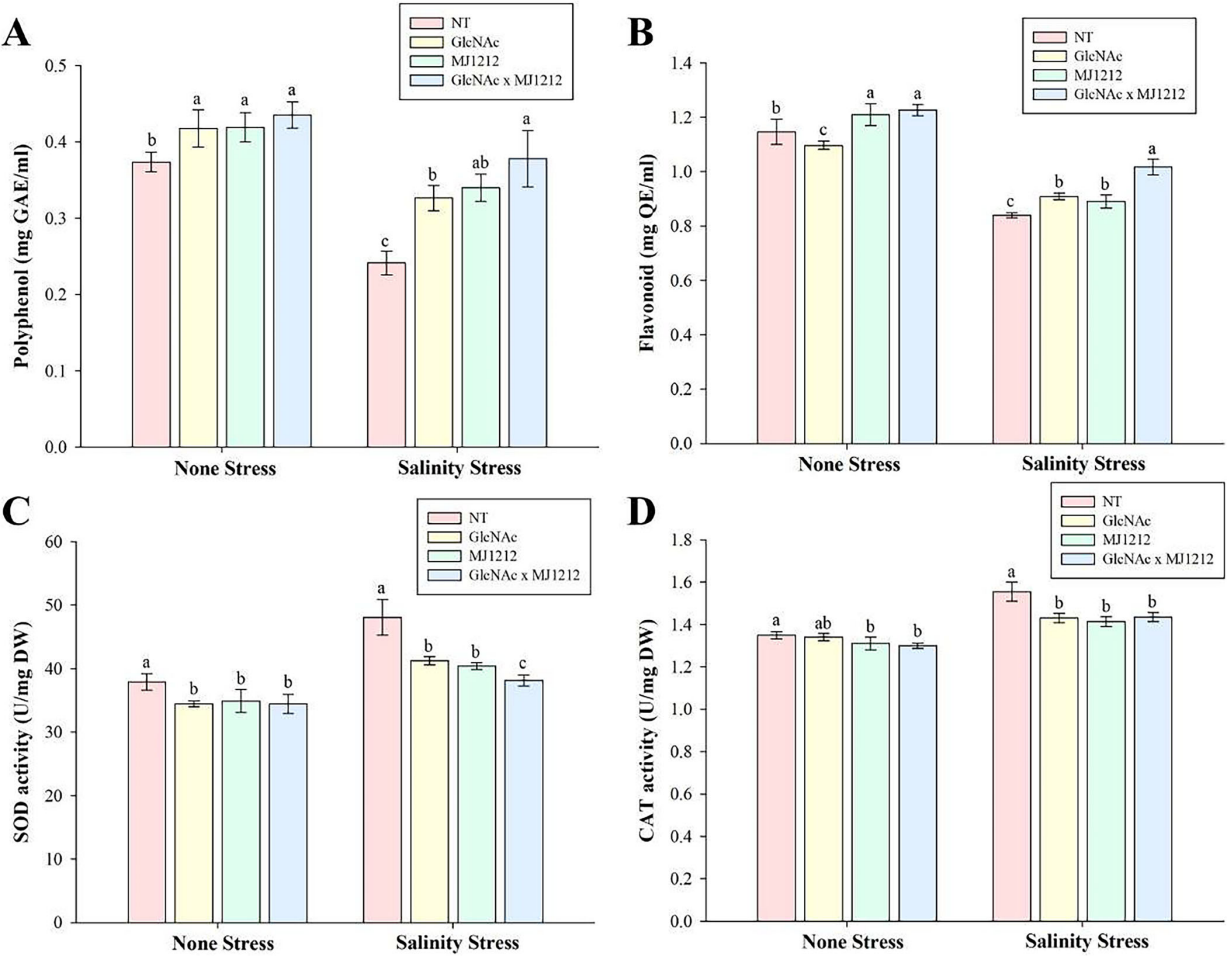
Effect of GlcNAc, *P. megaterium* mj1212 and their combination on (A) polyphenol content, (B) flavonoid content, (C) superoxide dismutase activity and (D) catalase activity under non‐stress and salinity stress conditions. Each bar represents the mean of three experimental replicates, with error bars indicating the standard error of the mean. Different letters above the bars indicate significant differences between treatments at *p* < 0.05, as determined by DMRT. [Color figure can be viewed at wileyonlinelibrary.com]

The results revealed statistically significant upregulation in the antioxidant activities of PPO and flavonoids in soybean plants treated with GlcNAc and mj1212, both under control and NaCl stress conditions. In the individual treatments, mj1212 showed a greater effect than GlcNAc, increasing PPO levels by 12.17% and 40.7% under control and stress conditions, respectively. The combined application of GlcNAc and mj1212 showed a synergistic effect, increasing PPO levels by 16.48% and 56.52% under control and stress conditions, respectively, compared to their non‐treated counterparts (Figure 5A). Similarly, flavonoid content increased in soybean plants treated with GlcNAc and mj1212, both individually and in combination. Compared to GlcNAc, mj1212 inoculation led to a greater increase in flavonoid content, enhancing it by 5.5% and 6.06% under control and NaCl stress conditions, respectively. This increase was further amplified when GlcNAc and mj1212 were applied together. However, under control conditions, individual treatment of GlcNAc resulted in a 4.3% reduction in flavonoid content (Figure 5B). Though NaCl stress significantly increased SOD and CAT activities, their levels were significantly reduced by the application of GLcNAc and mj1212. The individual application of GlcNAc and mj1212 resulted in a 9.02% and 7.91% decrease in SOD content under non‐stress conditions, respectively, with a similar trend observed in plants treated with mj1212 alone and those treated with both GlcNAc and mj1212. Under NaCl stress, the combined application of GlcNAc and mj1212 significantly reduced the SOD level by 20.7% (Figure 5C). A similar trend was observed for CAT concentrations, which were higher in NaCl‐stressed samples (Figure 5D).

### 3.7 Effects of GlcNAc and mj1212 on OAs Activities Under NaCl Stress

**Figure 6:**
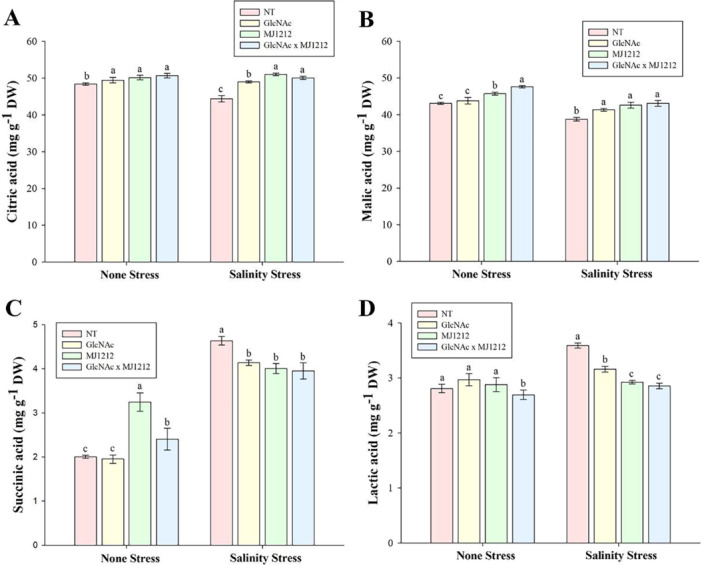
Effect of GlcNAc, *P. megaterium* mj1212 and their combination on (A) citric acid, (B) malic acid, (C) succinic acid and (D) lactic acid under non‐stress and salinity stress conditions. Each bar represents the mean of three experimental replicates, with error bars indicating the standard error of the mean. Different letters above the bars indicate significant differences between treatments at *p* < 0.05, as determined by DMRT. [Color figure can be viewed at wileyonlinelibrary.com]

To further understand the mitigation of salt stress through the application of GlcNAc and mj1212, we evaluated the levels of OAs. Both individual and combined applications of GlcNAc and mj1212 increased CAs and MAs levels under both control and stress conditions. Plants inoculated with mj1212 exhibited higher CAs levels (3.5%) compared to those treated with GlcNAc (2.12%), with an increase of up to 14.88% under NaCl stress. However, under stress conditions, the combined application of GlcNAc and mj1212 had an antagonistic effect, increasing CAs levels by only 12.74%, which was lower than the individual treatment with mj1212 (Figure 6A). A similar trend was observed for MAs, however in this case, higher MAs levels were found in control plants compared to stressed plants, with the highest increase (10.54%) observed in plants treated with both GlcNAc and mj1212 (Figure 6B). The concentration of SAs was very low in sole‐watered plants but significantly increased with both individual and combined applications of mj1212 and GlcNAc. Under stress conditions, SAs concentration was significantly higher in sole‐NaCl‐treated plants, but both individual and combined applications of GlcNAc and mj1212 led to a significant reduction. For example, the combined application of GlcNAc and mj1212 decreased SAs levels by 14.88% compared to sole‐NaCl treated plants (Figure 6C). In contrast to SAs, LAs concentration decreased significantly by 4.27% and 20.33% when plants were treated with GlcNAc and mj1212 together under control and NaCl stress conditions, respectively (Figure 6D).

### 3.8 Effects of GlcNAc and mj1212 on ABA Content Under NaCl Stress

**Figure 7:**
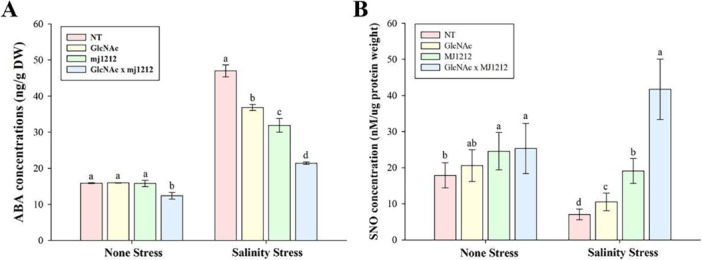
Effect of GlcNAc, *P. megaterium* mj1212 and their combination on (A) endogenous abscisic acid and (B) S‐Nitrosothiol level under non‐stress and salinity stress conditions. Each bar represents the mean of three experimental replicates, with error bars indicating the standard error of the mean. Different letters above the bars indicate significant differences between treatments at *p* < 0.05, as determined by DMRT. [Color figure can be viewed at wileyonlinelibrary.com]

The results indicated that, under non‐stress conditions, the individual treatment with GlcNAc and mj1212 had no significant effect on ABA concentration. However, their combined application significantly reduced the ABA level by 22.16% compared to control plants. The highest ABA level (195.48%) was recorded in plants exposed to 150 mM NaCl stress compared to control plants. Individual treatments with GlcNAc and mj1212 reduced ABA levels by 21.63% and 32.07%, respectively. The combined application of GlcNAc and mj1212 showed a synergistic effect, decreasing the ABA level by 54.4% compared to NaCl‐treated plants (Figure 7A).

### 3.9 Effects of GlcNAc and mj1212 on SNO Concentration Under NaCl Stress

SNO concentration varied significantly between non‐stress and NaCl stress conditions in response to different treatments. Under non‐stress conditions, sole‐watered plants recorded a baseline value of 17.86, which increased by 15.15% with GlcNAc, 37.5% with mj1212 and 41.82% with the combined application of GlcNAc and mj1212. Under NaCl stress, sole‐NaCl‐treated plants showed a 60.59% decline in SNO concentration compared to non‐stressed, sole‐watered plants. However, GlcNAc application increased the concentration by 49.35%, while mj1212 enhanced it by 170.97%. The highest recovery was observed with the combined application of GlcNAc and mj1212, resulting in a 492.55% increase compared to sole‐NaCl‐treated plants (Figure 7B).

## 4 Discussion

Addressing the adverse impacts of salt stress on crops in agricultural fields is a significant challenge for sustainable agriculture. Although various technological approaches have been developed to reduce salt‐induced damage, many of these solutions are costly, energy‐demanding and pose potential long‐term ecological risks. Therefore, biological solutions, such as PGPR and organic amendments, provide a more sustainable and effective alternative (Woo et al. 2025). The current study explored the combined application of *P. megaterium* (mj1212) and GlcNAc to alleviate salt stress in soybean plants, focusing on physiological and biochemical perspectives. Our previous studies demonstrated that mj1212 exhibited PGP traits, such as metabolite production, phosphate solubilisation, enhanced carbohydrate and amino acid contents, modulation of the antioxidant system and regulation of endogenous ABA biosynthesis (Kang et al. 2021; Kang et al. 2014). CAZy database analysis revealed that the genome of mj1212 strain encodes various enzymes involved in carbohydrate synthesis, metabolism and transport, which are vital for the strain's survival and adaptability. Metabolic analysis further helps in understanding the ecological roles of these enzymes and provides valuable insights for both basic and applied research, facilitating further exploration of their biological characteristics and potential applications (Zhang et al. 2024).

In the current study, we report complete genome sequencing and functional analysis of mj1212. Our results support the recent taxonomic reclassification of *P. megaterium* (J. M. Liu et al. 2023; Patel and Gupta 2020), placing the mj1212 strain within the genus Priestia and identifying it as *P. megaterium*. The presence of diverse BGCs, including both conserved and divergent types, indicates the strain's potential for producing a variety of secondary metabolites and suggests promising opportunities for the discovery of novel bioactive compounds. Furthermore, the identification of genes associated with PGPR traits reflects its functional adaptability and relevance in promoting plant health through mechanisms such as nutrient metabolism, stress tolerance and phytohormones regulation. Recently, *P. megaterium* species have been studied for the presence of genes associated with responses to environmental stresses such as salinity, temperature fluctuations and heavy metals exposure (Thakur et al. 2024). Specifically, the mj1212 genome encodes phosphate transport genes (*pstB*, *pstA*, *pstC*, *pstS*) important for phosphate metabolism (Cox et al. 1989; Lamarche et al. 2008), tryptophan‐related genes (*trpS*, *solA*, *kynA*, *trpA*, *trpB*) involved in IAA production (Suárez Pérez 2020), and osmoprotectant‐related genes (*proV*, *glycine betaine ABC transporter*, *betB*) supporting glycine betaine and proline transport (Choquet et al. 2005; Scholz et al. 2016). It also includes siderophore‐related genes for iron acquisition and a robust set of chemotaxis and motility genes (*MCPs*, *CheW*, *fli* family) facilitating root colonisation (Arnold et al. 2011). Together, these genomic features help to reduced ABA accumulation, modulate antioxidant activities and improved salt stress tolerance observed in soybean, promoting hormonal balance, nutrient uptake and redox homoeostasis for better stress resilience. In this study, mj1212 was found to modulate the antioxidant system, increase the production of various OAs, and influence phytohormones such as ABA, promoting soybean growth under salt stress. These findings are consistent with previous studies, which have demonstrated that *Bacillus spp*. have the potential to influence OAs production, and tolerate oxidative stress, thereby promoting soybean growth by modulating the production of ABA and IAA (Egamberdieva et al. 2017; Park et al. 2017). Similar effects have been reported in other crops where PGPMs inoculation enhanced salt tolerance by boosting antioxidant activity, phytohormones balance and nutrient uptake (B. Ali et al. 2022; Latif et al. 2024; Shabaan et al. 2022). To effectively mitigate salt stress, we applied GlcNAc in combination with mj1212. Our results revealed that, in most assays, their co‐application exhibited synergistic effects, significantly promoting soybean growth under salt stress. Various studies have demonstrated that salt stress suppresses soybean growth (Kang et al. 2023; M. Khan et al. 2021; Kwon et al. 2024). Our study also revealed that NaCl stress significantly reduced soybean plants growth. However, the application of GlcNAc and mj1212 effectively mitigated the adverse effects of NaCl by promoting shoot and root length as well as biomass accumulation. Notably, mj1212 exhibited a more pronounced effect than GlcNAc in enhancing these growth parameters, suggesting that Bacillus inoculation contributed to the production of osmoprotectants, induction of antioxidant defence systems and improved water retention, as previously demonstrated in soybean and other crops under salinity stress (Hasanuzzaman et al. 2022; Ji et al. 2022). Similarly, previous studies have shown that the co‐application of compost and the PGP bacterium *Azospirillum brasilense* in maize plants enhances nutrient uptake, strengthens antioxidant defences and improves soil structure under saline conditions (El‐Akhdar et al. 2025). These studies showed that the combined application of PGPMs and organic amendments such as compost and humic acids improves salt tolerance more effectively compared to individual treatments by synergistically enhancing plant‐microbial interactions and improving soil health (Abdelrahman et al. 2021; Priya et al. 2024). Extending these findings, the present study suggests that the synergistic effect observed with the combined application of GlcNAc and mj1212 may result from the complementary mechanisms, where GlcNAc‐induced signalling and mj1212‐mediated microbial interactions work together to enhance salt tolerance, as supported by previous studies (Naseem et al. 2012; You et al. 2024). Unlike previous studies, our study demonstrates that mj1212 combined with GlcNAc enhances salt tolerance through distinct mechanisms, including modulation of antioxidant activities, OAs metabolism, NO signalling and ABA regulation. Furthermore, the complete genome sequencing and functional annotation of mj1212 provide molecular evidence supporting these unique modes of action.

The results indicate that the individual treatment of GlcNAc significantly improved chlorophyll content compared to the individual application of mj1212 and its combination with GlcNAc. In addition, the adverse effects of NaCl stress on chlorophyll content were effectively mitigated by the combined application of GlcNAc and mj1212, which is consistent with previous studies on the role of organic amendments combined with PGP bacteria in improving chlorophyll contents under stress conditions (Ferdous et al. 2018; Ullah et al. 2021). Similar observations were reported by Alinia et al. (2022), Arshad et al. (2024) and Omara et al. (2022), showing that the co‐application of microbial inoculants and organic materials improved photosynthetic traits and plant growth under saline conditions in crops such as wheat and common bean. GlcNAc treatment increased electron transport flux and PSⅡ efficiency, while mj1212 increased photon absorption but reduced energy flux. Under stress conditions, the significant improvement in PSⅡ closure, photosynthetic rate and transpiration rate with the combined application of GlcNAc and mj1212 suggesting the improved energy efficiency, enhanced nutrient uptake, increased osmotic regulation and reduced oxidative damage. These findings align with previous studies indicating that exogenous treatment with silicon (Si) can boost photosynthetic performance of plants by enhancing photosystem function and mitigating oxidative stress (Malik et al. 2021). The RWC measurements showed that both GlcNAc and mj1212 significantly improved soybean plants’ turgor potential and water retention, supporting findings from previous studies that indicate the beneficial role of GlcNAc and bacterial strains in enhancing RWC in plants under salt stress conditions (Kang et al. 2024; Soliman et al. 2024).

The results revealed that the application of GlcNAc and mj1212 enhances the plant's antioxidative defence system and improves salt tolerance in soybeans by upregulating the expression of PPO and flavonoids. On the other hand, the reduced expression of antioxidative enzymes such as SOD and CAT suggests that these treatments mitigate ROS generation, thereby decreasing the reliance on enzymatic scavenging. These findings confirm previous studies showing that beneficial microbes positively influence the balance of antioxidant enzymes to detoxify ROS (I. Khan et al. 2025; Kwon et al. 2024). Similarly, (Arora et al. 2024; Kavian et al. 2023) reported that beneficial microbial inoculants combined with organic amendments modulated ROS‐scavenging pathways, leading to enhanced oxidative stress tolerance in crops such as rice and maize.

The modulation of the plant's antioxidant system to reduce ROS may be associated with changes in the release of OAs such as CAs, MAs, SAs and LAs. These OAs are intermediates in the TCA cycle, a vital metabolic pathway that generates energy and contributes to antioxidant production. Previous studies have shown that both external application and natural release of OAs help plants cope with excessive ROS induced by environmental stresses, including salt stress (Bilal et al. 2022). In the current study, the increase in CAs and MAs with GlcNAc and mj1212 under salt stress is possibly due to enhanced TCA cycle activity, ROS detoxification and improved ion homoeostasis, while the decrease in SAs and LAs suggests increased energy efficiency and a metabolic shift towards stress adaptation, as supported by previous studies (H. Lee et al. 2023; Tahjib‐Ul‐Arif et al. 2021). The synergistic effects of GlcNAc and mj1212 likely result from GlcNAc‐induced signalling pathways that prime cellular metabolism, in combination with the PGP traits of mj1212, collectively amplifying carbon flux through the TCA cycle. This interaction enhances both energy production and the availability of precursors for antioxidant synthesis, helping maintenance of metabolic balance under salt stress. Similar shifts in OA profiles and TCA cycle adjustments have been observed in plants treated with PGPMs and organic amendments, indicating a broader, conserved mechanism for enhancing salt tolerance across species (Woo et al. 2025>; Zainurin et al. 2025).

With the application of GlcNAc and mj1212, SNO concentrations increased under both control and stress conditions. These elevated SNO levels may promote S‐nitrosylation, a post‐translational modification in which NO binds to cysteine residues in proteins, influencing their stability, interactions and function. This modification is essential for regulating enzymes, signal transduction and responses to oxidative stress (D.‐S. Lee et al. 2024). However, it is important to note that dysregulated or excessive S‐nitrosylation can have deleterious effects. Over‐nitrosylation may lead to protein dysfunction, inhibition of enzyme activity and interference with normal cellular signalling, particularly under nitrosative stress conditions (Gu et al. 2010). Moreover, excessive NO levels can contribute to redox imbalance and oxidative damage by interacting with ROS, leading to the formation of peroxynitrite (ONOO^−^) and other reactive intermediates such as NO_2_, N_2_O_3_ and various NO_x_ compounds (Lindermayr and Durner 2015). These considerations emphasise the importance of maintaining tightly regulated NO homoeostasis within cells, as the beneficial effects of mj1212‐GlcNAc‐induced S‐nitrosylation may be accompanied by potential risks, including oxidative or nitrosative stress. Therefore, further studies are required to comprehensively elucidate these effects and ensure a balanced physiological response.

This study revealed a significant increase in endogenous ABA content in soybean plants under NaCl. This finding is consistent with previous studies showing that salt stress induces ABA biosynthesis as an adoptive mechanism, regulating stomatal closure and minimising water loss to counteract salt‐induced drought stress (Asif et al. 2023; Lubna et al. 2022). Application of GlcNAc and mj1212 significantly reduced ABA accumulation, with a further reduction observed when plants were treated with both GlcNAc and mj1212 in combination. This reduction may help prevent stress‐induced growth inhibition, as excessive ABA levels can negatively affect plant growth and development (Brookbank et al. 2021; D. Feng et al. 2024). There is a potential cross‐talk between endogenous ABA and enzymatic antioxidants such as SOD in plant defence responses. SOD plays a crucial role by catalysing the conversion of superoxide radicals (O_2_^−^) into molecular oxygen and H_2_O_2_, thereby alleviating oxidative stress (I. Khan et al. 2025). This interaction indicates that ABA, modulated by GlcNAc and mj1212, potentially plays a key role in regulating antioxidant defences under salt stress, thereby enhancing the stress tolerance of soybean plants.

In summary, this study demonstrates that the application of GlcNAc and MJ1212 enhances salt stress tolerance in soybean by modulating various physiological, biochemical and molecular mechanisms. These findings provide a novel research direction for plant genome modification and the development of crop varieties capable of remediating salt‐contaminated soils and alleviating salinity impacts on agricultural systems, contributing to food and environmental safety. However, it is important to note that these findings were obtained under controlled environment conditions, which may not fully reflect the complexity of field environments. Therefore, further studies and field trials are needed to evaluate the practical effectiveness and adaptability of these approaches under real‐world agricultural conditions.

## 5 Conclusion

The ever‐growing demand for food, coupled with the dramatic increase in environmental pollution, including salt stress, underscores the urgent need for the adaptation of sustainable agricultural practices that enhance productivity and promote eco‐friendly approaches. The current study provides invaluable insights into practical strategies for sustainable agriculture, particularly in salt stress mitigation through the application of beneficial microbes with organic amendments. Key findings include the whole genome sequencing and annotation of the mj1212 bacterial strain, which revealed PGP and stress tolerance traits, as well as the practical effects of mj1212 and GlcNAc on improving soybean growth, photosynthetic efficiency, antioxidants activity, important OAs levels, SNO concentration and ABA content under salt stress. Furthermore, the study demonstrates that OAs effectively modulate energy metabolism and stress adaptation, while SNO actively enhance signalling pathways to improve plant resilience under salt stress. While this study focused primarily on soybean plants, the insights gained extend more broadly to developing eco‐friendly agricultural practices, offering practical implications for improving crop resilience and ensuring food security under saline conditions. Future research should focus on field validation to assess the practical effectiveness and scalability of mj1212‐GlcNAc treatments under real agricultural conditions. In addition, unravelling the molecular and biochemical pathways underlying mj1212‐GlcNAc interactions in salt stress mitigation is essential to deepen our mechanistic understanding and support the development of precision microbial solutions for sustainable agriculture.

## Conflicts of Interest

The authors declare no conflicts of interest.

## Supporting information


**Figure S1:** Growth of *P. megaterium* MJ1212 in response to different GlcNAc concentrations on TSB medium at 28 °C, cultured for 48 hr in a shaking incubator at 150 rpm.

**Figure S2:** Effect of GlcNAc, *P. megaterium* mj1212, and their combination on relative water contents of soybean plants under none‐stress and salinity stress conditions. Each bar represents the mean of three experimental replicates, with error bars indicating the standard error of the mean. Different letters above the bars indicate significant differences between treatments at p < 0.05, as determined by DMRT.

**Table S1:** Genome features of P. megaterium mj1212.

**Table S2:** Genes associated with PGPR traits in the genomes of *P. megaterium* mj1212.

**Table S3:** COG categories detail.

**Table S4:** CAZyme families, gene counts, and their functional roles.

**Table S5:** Description of chlorophyll fluorescence parameters derived from the OJIP test, representing various aspects of PSII photochemistry and energy fluxes.

## Data Availability

The data that support the findings of this study are available on request from the corresponding author. The data are not publicly available due to privacy or ethical restrictions.
