# Supplementary figures and images for: Genome Characterisation of Priestia megaterium mj1212 and Its Synergistic Effect With N‐Acetylglucosamine in Enhancing Soybean Salt Stress Tolerance
Source: Plant Cell Environ. 2025 Aug 5;48(11):8006–23. doi: 10.1111/pce.70093 (PMC12502018; doi:10.1111/pce.70093)

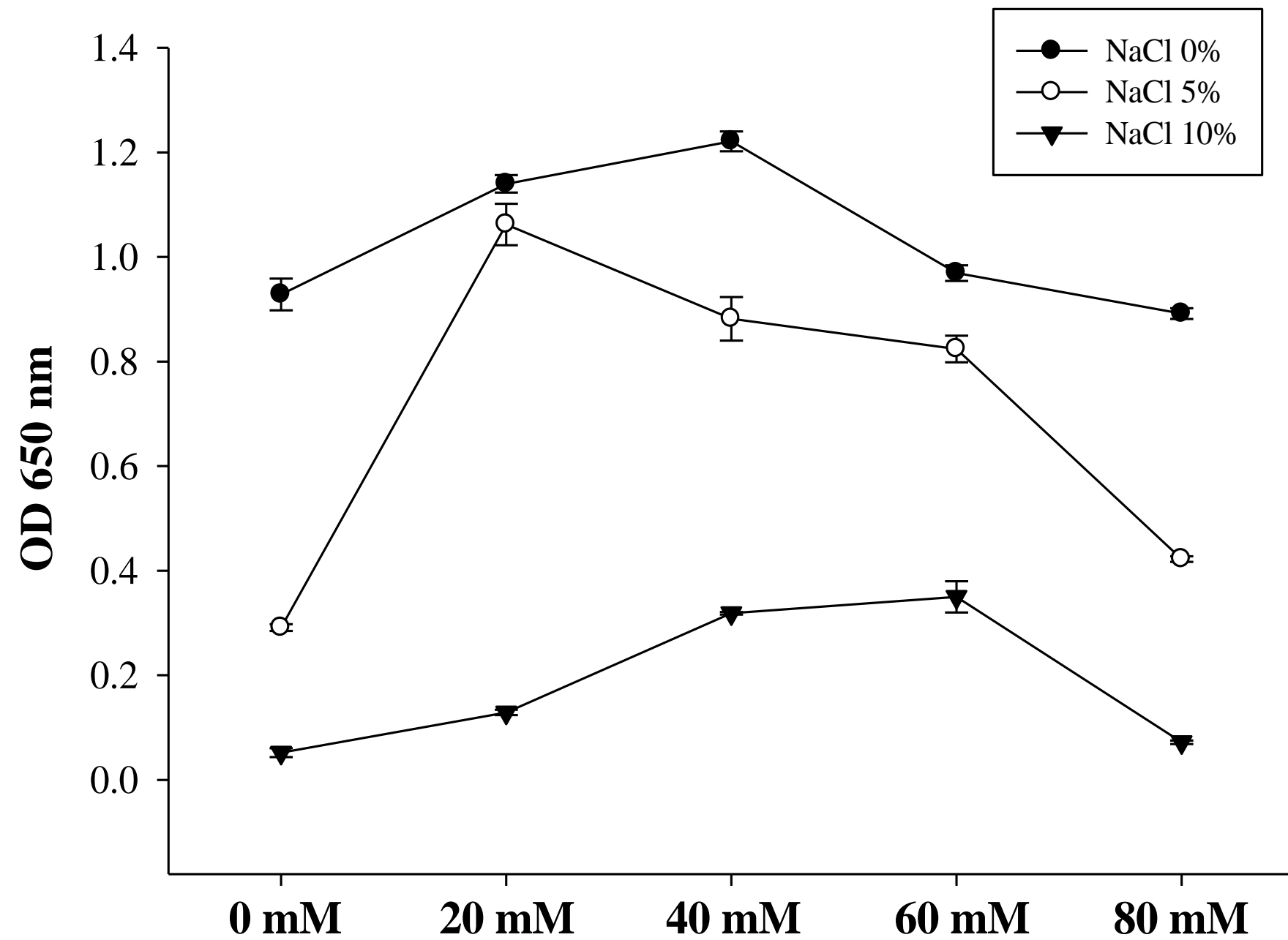

Supplement: Supplementary file 1 — Figure S1: Growth of P. megaterium MJ1212 in response to different GlcNAc concentrations on TSB medium at 28 °C, cultured for 48 hr in a shaking incubator at 150 rpm. [file PCE-48-8006-s001.pdf]

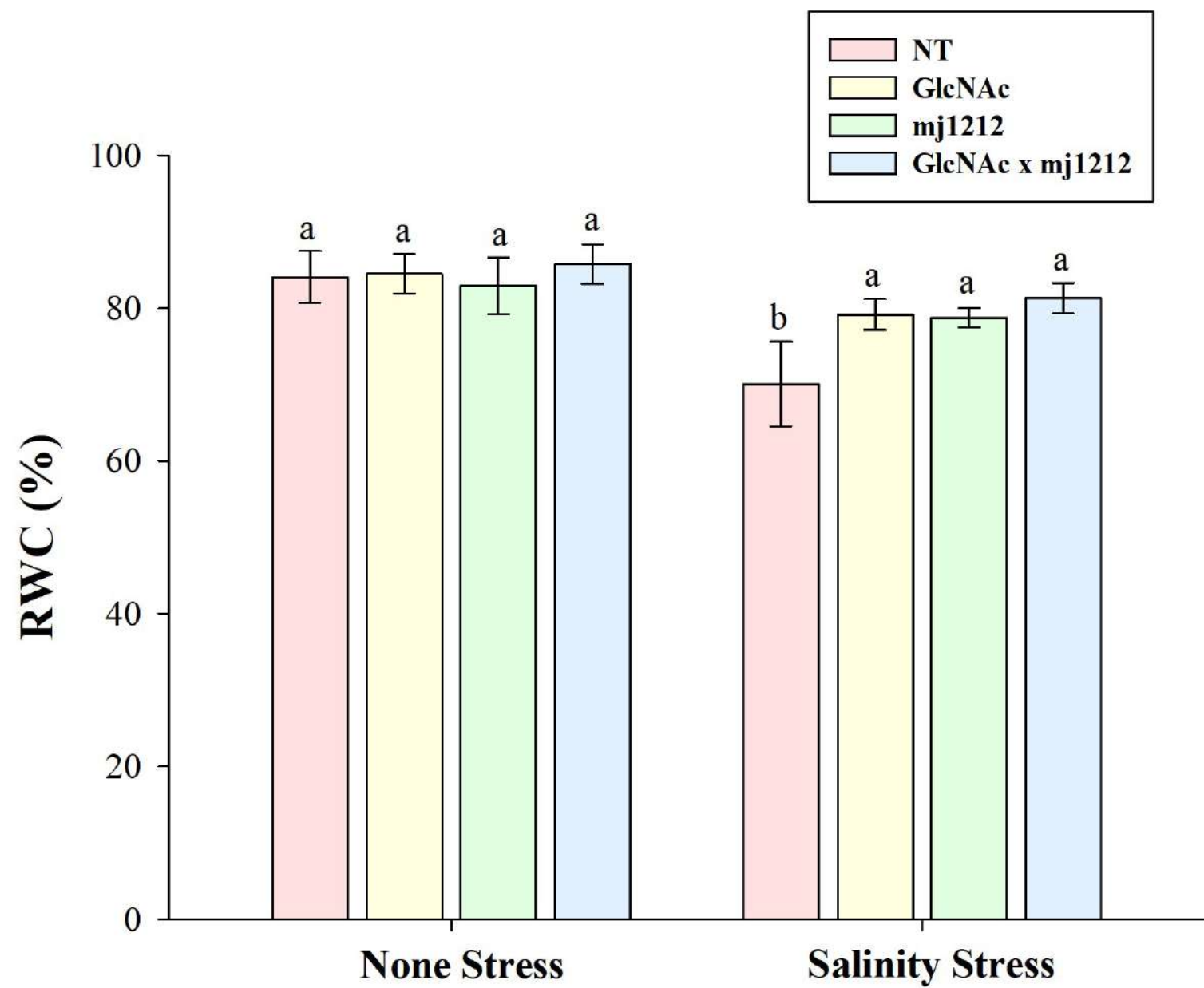

Supplement: Supplementary file 2 — Figure S2: Effect of GlcNAc, P. megaterium mj1212, and their combination on relative water contents of soybean plants under none‐stress and salinity stress conditions. Each bar represents the mean of three experimental replicates, with error bars indicating the standard error of the mean. Different letters above the bars indicate significant differences between treatments at p < 0.05, as determined by DMRT. [file PCE-48-8006-s006.pdf]
