# Supplementary material for: Genome Characterisation of Priestia megaterium mj1212 and Its Synergistic Effect With N‐Acetylglucosamine in Enhancing Soybean Salt Stress Tolerance
Source: Plant Cell Environ. 2025 Aug 5;48(11):8006–23. doi: 10.1111/pce.70093 (PMC12502018; doi:10.1111/pce.70093)
Supplement: Supplementary file 7 — Table S5: Description of chlorophyll fluorescence parameters derived from the OJIP test, representing various aspects of PSII photochemistry and energy fluxes. [file PCE-48-8006-s005.docx]

| **Parameter** | **Description** |
| --- | --- |
| **Vj** | Relative variable fluorescence at 2 ms |
| **M_o_** | Net rate of PSII closure |
| **F_v_/F_o_** | Efficiency of the water-splitting complex on the donor side of PSII |
| **ABS/RC** | Absorbed photon flux per PSII reaction center |
| **TR_o_/RC** | Trapped energy flux per PSII reaction center |
| **DI_o_/CS** | Dissipation energy flux per cross-section |
| **ET_o_/RC** | Electron transport flux from QA to QB per PSII reaction center |
| **ET_o_/CS** | Electron transport flux from QA to QB per cross-section |
| **DI_o_/RC** | Dissipation energy flux per PSII reaction center |
